# Suppressive Effects of D-Glucosamine on the 5-HT Sensitive Nociceptive Units in the Rat Tooth Pulpal Nerve

**DOI:** 10.1155/2014/187989

**Published:** 2014-04-13

**Authors:** Kei Kaida, Hiromi Yamashita, Kazuo Toda, Yoshihiko Hayashi

**Affiliations:** ^1^Department of Cariology, Nagasaki University Graduate School of Biomedical Sciences, Sakamoto 1-7-1, Nagasaki 852-8588, Japan; ^2^Department of Integrative Sensory Physiology, Nagasaki University Graduate School of Biomedical Sciences, Sakamoto 1-7-1, Nagasaki 852-8588, Japan; ^3^Department of Regenerative Oral Surgery, Nagasaki University Graduate School of Biomedical Sciences, Sakamoto 1-7-1, Nagasaki 852-8588, Japan

## Abstract

It is well known that D-glucosamine hydrochloride (DGL) has a variety of biological activities and is regarded as a nutritional supplement effective in improving various disorders, including osteoarthritis and atherosclerosis. Although it has been reported that DGL has a significant pain relief effect in treating osteoarthritis, little is known about the characteristics of the effects of this compound on dental pain. The present study was undertaken to evaluate the applicability of DGL as a medicament to control pulpalgia. Using an *in vitro* rat mandible-inferior alveolar nerve preparation (jaw-nerve preparation), we evaluated the effects of DGL on 5-hydroxytryptamine (5-HT) sensitive nociceptive responses in the tooth pulpal nerve. 5-HT-induced nociceptive responses were fairly suppressed by direct application of DGL, suggesting that DGL have a pain relief effect on patients with dental pain.

## 1. Introduction

Chitin is a polysaccharide mainly located in the outer skeletal tissue of crustacean such as crabs, shrimps, and lobsters [1] and is the main source of production of chitosan. D-Glucosamine hydrochloride (DGL) is industrially produced by completely hydrolyzing chitin with hydrochloride.

DGL has a variety of biological activities. DGL suppress the functions of the neutrophils, thereby possibly exhibiting anti-inflammatory actions [2], and DGL is reported to suppress the progression of adjuvant arthritis [3]. Furthermore, DGL is able to suppress platelet aggregation [4] and synoviocytes activation (such as nitric oxide-, PGE_2_-, and IL-8-production and phosphorylation of p38 MAPK) [5].

DGL has been clinically used as an effective medicament [6, 7]. For example, DGL exhibits a significant antipain effect in treating osteoarthritis problems [8–12]. Therefore, DGL is widely used in an attempt to suppress the pain associated with disability of osteoarthritis [13]. Osteoarthritis is a disease with low expectations regarding the value of treatment [14, 15]. Nonsteroidal anti-inflammatory drugs (NSAIDs) are commonly used to treat osteoarthritis; however, they have major adverse effects [16] and may even worsen the osteoarthritic symptoms [17]. Several short- and long-term clinical trials of patients with osteoarthritis have shown the significant symptom-modifying effect of DGL [18–20].

We have shown that DGL accelerates cell proliferation and differentiation and promotes tissue regeneration on dental pulp wounds [21]. DGL suppressed interleukin-8 secretion from pulp fibroblast and DGL produced the initial anti-inflammatory reaction in the pulp tissue [21].

Previously, we used bradykinin as a chemical nociceptive stimulant and evaluated the possibility of DGL as a medicament to control dental pain. The results suggested that DGL have a pain relief effect on patients with dental pain.

However, the pain relief mechanism of DGL in the setting of dental pain is poorly understood. In this study, we revaluated whether DGL exhibits relief of dental pain caused by the pain-producing substance, 5-hydroxytryptamine (5-HT), instead of bradykinin in order to elucidate the pain relief of mechanism of DGL.

## 2. Materials and Methods 

The methods described here follow the ethical guidelines of and received approval from the Animal Welfare Committee of Nagasaki University (number 0806090666, 2008–2011).

### 2.1. Preparation

**Figure 1:**
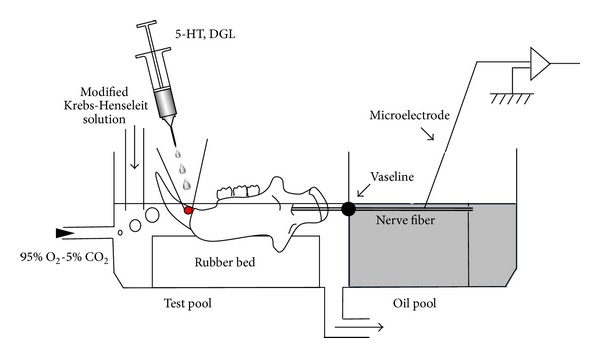
Schematic presentation of the chamber setup for the* in vitro* jaw-nerve preparation (see text for details).

Twenty male Wistar albino rats (body weight: approximately 200 g) were used in the present study. The animals were deeply anesthetized with thiamylal sodium (60 mg/kg, i.p., Isozol, Nippon-Iko Pharmacy, Toyama, Japan). We used an* in vitro* jaw-nerve preparation, as described elsewhere [22]. An original* in vitro* jaw-nerve preparation was slightly modified in order to enable easy direct access to the dental pulp (Figure 1). The mandible was divided into the right and left halves at the central suture using a pair of scissors, and the surrounding masticatory muscles, including the masseter, temporalis, zygomaticus, and mediolateral pterygoid muscles, were completely removed using surgical scissors.

The inferior alveolar nerve was identified at the foramen mandibulae on one side and isolated from the surrounding tissue. The mandible on one side was removed together with the inferior alveolar nerve by cutting the temporomandibular joint. Finally, approximately 20 mm of the inferior alveolar nerve trunk was obtained from the foramen mandibulae and the proximal end of the nerve was ligated with cotton thread. A small hole was carefully made in the center of the incisor tooth in order to expose the dental pulp using a dental bur (round bur number 3, Meisinger, Neuss, Germany).

### 2.2. Drug Solutions

#### 2.2.1. Modified Krebs-Henseleit Solution

Modified Krebs-Henseleit solution consisting of 110.9 mM of NaCl, 4.8 mM of KCl, 2.5 mM of CaCl_2_, 1.2 mM of MgSO_4_, 1.2 mM of KH_2_PO_4_, 22.4 mM of NaHCO_3_, and 20 mM of glucose was used for perfusion.

#### 2.2.2. 5-HT Solution

5-HT (C_10_H_12_N_2_O) (Sigma, St. Louis, MO, USA) was dissolved in the sterile physiological saline (Otsuka Pharmaceutical Co., Tokyo, Japan). 10^−5^ M 5-HT solution was used to chemically stimulate the tooth pulpal nociceptors.

#### 2.2.3. DGL Solution

DGL was supplied by Koyo Chemical Co., (Osaka, Japan). The molecular weight of the DGL used in this experiment was approximately 215 Da. A 10% (w/v) solusion was prepared by dissolving DGL powder in sterile physiological saline.

### 2.3. Chamber Design

As shown in Figure 1, a chamber (total volume: 147 mL) was made using a plastic plate (thickness: 2 mm). The chamber room was separated into two pools (test pool: 84 mL; oil pool: 63 mL) using a thin plastic plate (thickness: 1 mm), at the center of which a small hole (diameter: 1.5 mm) was drilled to pass the trunk of the inferior alveolar nerve. A hard rubber bed was attached to the bottom of the test pool in order to prevent damage to the preparation.

### 2.4. Recording Procedures

The mandible was placed in the test pool, and the inferior alveolar nerve was passed through a hole at the center of the partition plate and placed in the recording chamber (oil pool), where it was fixed to the wall of the chamber with cotton thread. A moderate amount of tension was applied so as to easily insert the recording electrode. The hole at the center of the partition plate was capped with Vaseline. The oil pool was filled with liquid paraffin in order to immerse the inferior alveolar nerve. The test pool was perfused (0.3 mL/s) with the modified Krebs-Henseleit solution. The solution was saturated with a gas mixture of O_2_ : CO_2_ (95% : 5%). The fluid was heated to maintain the temperature at 31°C with a feedback-controller.

The inferior alveolar nerve in the oil pool was desheathed by slipping off the epineurium. A tungsten microelectrode (tip impedance 10–12  MΩ at 10 kHz, A-M systems, Carlsborg, Washington, USA) was inserted into the inferior alveolar nerve trunk using a micromanipulator (MP-1, Narishige, Tokyo, Japan) in order to record single fiber responses. The action potential was fed into a high-impedance, low-noise amplifier (DAM-80, WPI Instruments, New Haven, CT, USA) and displayed on a computer through the CED 1401 interface (Cambridge Electronic Design Limited, Cambridge, UK). A spike analysis was carried out using the Spike 2 software program for Windows (Version 2.01).

It is shown that C-fibers terminals are located in the center core of the pulp [23]. In order to test chemical nociceptive sensitivity in the pulpal nerve, we selected only C-fiber responses for a quantitative analysis and observed one unit per rat in order to prevent sensitization of the nociceptor. We analyzed each unit with firing rate below 2.0 Hz, as the spontaneous discharge of the C-fiber has a mean frequency of 1.6 ± 0.5 Hz (range: below 2.0 Hz) as reported by Xiao and Bennett [24].

5-HT was used as a chemical nociceptive stimulant and poured into the test chamber with application near the exposed tooth pulp at a speed of 0.1 mL/s. Sixty seconds after 5-HT application, the surface of the exposed pulp was treated with DGL solution in the DGL group and physiological saline in the control group.

### 2.5. Statistical Analysis

The obtained data were evaluated statistically using the StatView software program, Version 5.0 (SAS Institute Inc., Cary, NC). The values were expressed as the mean ± SE, and the differences between the control and DGL groups were compared using unpaired Student's *t*-test. A *P* value of less than 0.05 was considered to be significant.

## 3. Results

**Figure 2:**
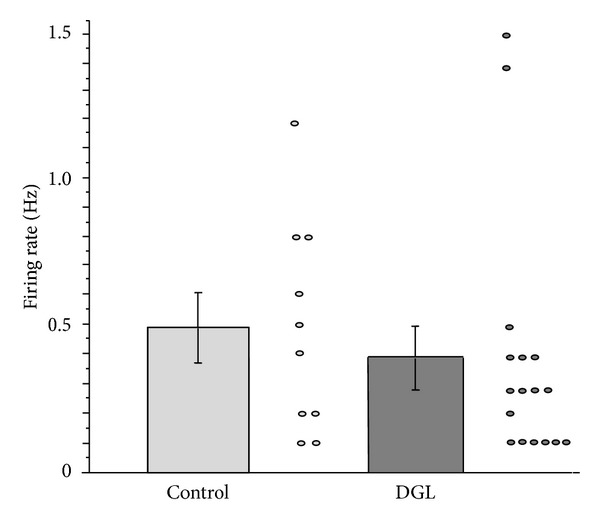
Firing rate (Hz) just after finishing making* in vitro* preparation in the control and DGL groups. There were no significant differences (*P* > 0.05). The dots indicate the firing rate, and the vertical bars denote the standard error.

A total of 20 single unit responses were obtained. Initially, a majority of the units spontaneously fired at 0.1–2.0 Hz. This activity was a good indicator that the magnitude of damage following surgical removal of the jaw was not too severe to obtain electrophysiological recordings. The firing rate just after preparation in the control group was 0.49 ± 0.12 Hz (*n* = 10), while that in the DGL group was 0.39 ± 0.10 Hz (*n* = 17). There were no significant differences in the initial firing rate before 5-HT application between the control and DGL groups (Figure 2).

**Figure 3:**
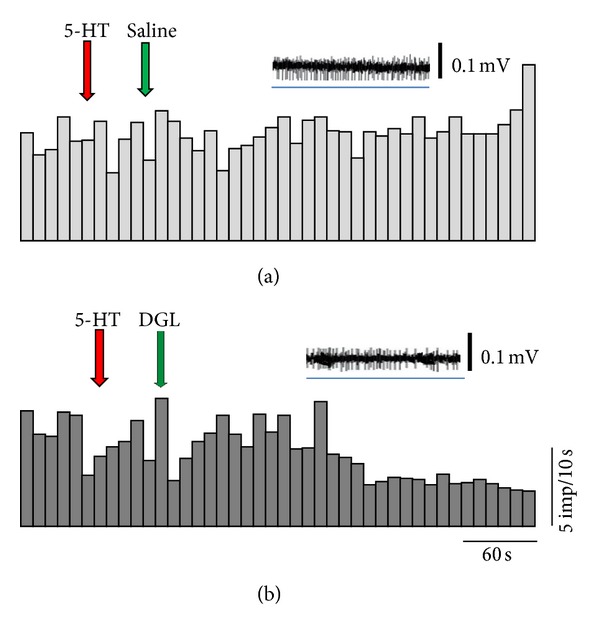
Typical examples of pulpal unit responses to 5-HT application and the effects of saline (a) and DGL (b) on the 5-HT-induced responses. The arrows indicate the time points of 5-HT (red arrows) and saline or DGL (green arrows) applications. Insets above the histograms show raw spikes during blue lines.

Figure 3 shows a typical example of the pulpal unit responses to 5-HT application and the effects of saline (a) and DGL (b) on the 5-HT-evoked responses. The histograms show the number of impulses/10 sec. The raw data above each histogram show responses after physiological saline and DGL application indicated by horizontal lines (blue). The arrows indicate the time of 5-HT (red) and DGL or saline (green) application, respectively. 5-HT application increased the spike responses, indicating that nociceptive responses were evoked in the tooth pulpal nerve. Following DGL application, the 5-HT-induced excitatory responses gradually decreased; however, saline application had no effects.

**Figure 4:**
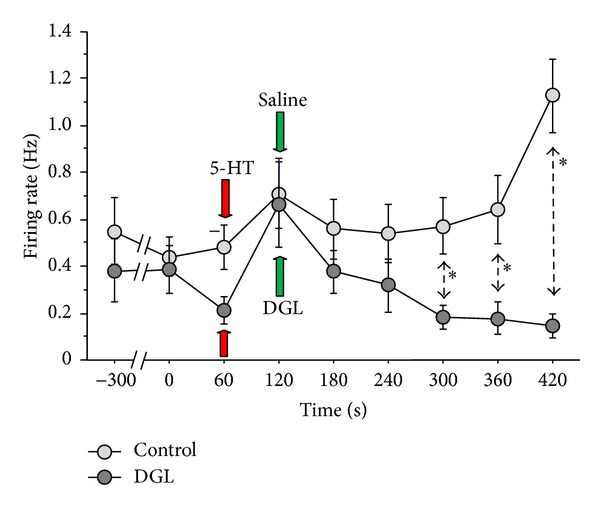
Effect of DGL on the 5-HT-induced responses. The arrows indicate the time points of 5-HT (red) and saline or DGL (green) applications. The DGL group exhibited a significantly lower frequency than the control groups after 300–420 seconds. The asterisks indicate significant differences, *P < 0.05. The vertical bars denote the standard error.

Figure 4 shows a summary of the time course of the effects of DGL on the 5-HT-evoked responses. The firing rate in the control group (light gray circle) continued to fire at state steady after saline application. While the time course changes in the frequency were not significantly different between the two groups prior to DGL or saline applications; the responses observed in the DGL group (dark gray circle) were decreased thereafter compared to those observed in the control group. The frequency in the control group was 0.92 ± 0.15 Hz (*n* = 10) five minutes after saline application, while that in the DGL group was 0.15 ± 0.05 Hz (*n* = 17) five minutes after DGL application. The DGL group exhibited a significantly lower frequency than the control groups (asterisk) during 300–420 s (*P* < 0.05).

## 4. Discussion

*In vitro* preparations have advantages in quantitatively controlling all environmental variables surrounding the oral tissue and directly applying chemical solutions to the receptive field [22]. Therefore, we investigated the analgesic effects of DGL on the tooth pulp “directly” although many previous clinical trials have evaluated the effects of DGL following oral administration [20, 25, 26]. The molecular weight of DGL is approximately 215, and this agent is thought to easily dissolve at the applied site although it may also easily make contact with the tooth pulp tissue as a dressing material.

We have investigated analgesic effect using 1, 5, 10, and 15% of DGL in preliminary study. In the present study, we used a 10% (w/v) solution prepared by dissolving DGL powder in sterile physiological saline. As a result, 10% DGL has been determined as an optimal concentration. At this concentration, there appeared to be no inflammatory reactions, as no enhancement of spike responses was observed after DGL application as shown in Figure 4.

The effects of DGL on dental pain have been little reported. Previously, we used bradykinin as a chemical nociceptive stimulant and to assess whether DGL exerts a pain relief effect in patients with dental pain [27].

The present study was undertaken in order to evaluate the effects of directly applying DGL to exposed dental pulp and demonstrated that DGL suppresses the nociceptive responses evoked by 5-HT application on the tooth pulp. These results indicate that DGL suppresses the nociceptive responses evoked by two different chemical nociceptive stimulants, namely, bradykinin and 5-HT.

TRPV1 is a cation channel that is activated by a variety of chemical stimulants. The best-known activator of TRPV1 is capsaicin. The activation of TRPV1 results in painful sensations. Bradykinin can sensitize TRPV1 via the G protein coupled receptor (GPCR) [28, 29]. Furthermore, the TRPV1 function is enhanced by 5-HT receptor activation [30]. It is possible that DGL may have an antinociceptive effect by binding to TRPV1.

In addition, it has been reported that DGL reduces the elementary current amplitude and increases the mean channel open time [31]. Because DGL has a weak binding site in the channel itself, the channel cannot be closed [31]. Voltage-gated sodium channels, which are necessary for electrogenesis and nerve impulse conduction, can be dynamically regulated after nerve injury or peripheral inflammation and can play important roles in modulating neural excitability [32, 33]. DGL may, thus, exhibit an antinociceptive effect by binding to sodium channels, resulting in a longer open time.

The precise mechanisms underlying the antinociceptive actions of DGL have not yet been elucidated; however, glucosamine-induced antinociception is presumed to be caused by anti-inflammatory effects. DGL suppresses neutrophil functions (such as chemotaxis, phagocytosis, superoxide generation, and granular enzyme release) [2] and inhibits the chronic phase of the inflammatory reaction and the production of inflammatory mediators (NO and PGE_2_) [3]. Therefore, glucosamine is strongly expected to be a potential novel anti-inflammatory agent.

As stated before, DGL has a significant pain relief effect in patients with osteoarthritis [34, 35]. Osteoarthritis is the most common joint disease and the leading cause of pain and physical disability in the elderly population. Elderly patients exhibit a high prevalence of multiple diseases that must be managed simultaneously and often require the use of different drugs. Conventional pharmacological approaches to symptom management in the setting of osteoarthritis involve the administration of nonsteroidal anti-inflammatory drugs. However, accumulating data show that many of these pharmaceutical drugs frequently produce insufficient benefits, with an associated risk of untoward side effects [36–38]. Glucosamine appears to be an attractive alternative, as it is a naturally occurring compound in the articular cartilage, while DGL is a symptom modifying drug with good evidence for favorable long-term effects on disease progression [39, 40]. DGL exerts a significant pain relief effect in treating osteoarthritis. Therefore, DGL is widely used in an attempt to suppress pain and treat disability in the setting of osteoarthritis.

The results of the present study show that DGL may have a pain relief effect in patients with dental pain. These findings led us to examine the application of DGL as a dental analgesic agent. The use of glucosamine to treat dental disorders is thought to be a possible alternative with a promising future.
